# Discordant definitions of small airway dysfunction between spirometry and parametric response mapping: the HRCT-based study

**DOI:** 10.1186/s13244-024-01819-0

**Published:** 2024-10-02

**Authors:** Bin Chen, Pan Gao, Yuling Yang, Zongjing Ma, Yingli Sun, Jinjuan Lu, Lin Qi, Ming Li

**Affiliations:** 1 Department of Radiology, Huadong Hospital Affiliated to Fudan University, Shanghai, China; 2 Zhang Guozhen Small pulmonary Nodules Diagnosis and Treatment Center, Shanghai, China; 3 Department of Radiology, Shanghai Geriatric Medical Center, Shanghai, China

**Keywords:** Computed tomography, Airways disease, Spirometer, Parametric response mapping

## Abstract

**Objectives:**

To analyze the lung structure of small airway dysfunction (SAD) defined by spirometry and parametric response mapping (PRM) using high-resolution computed tomography (HRCT), and to analyze the predictive factors for SAD.

**Methods:**

A prospective study was conducted with 388 participants undergoing pulmonary function test (PFT) and inspiratory-expiratory chest CT scans. The clinical data and HRCT assessments of SAD patients defined by both methods were compared. A prediction model for SAD was constructed based on logistic regression.

**Results:**

SAD was defined in 122 individuals by spirometry and 158 by PRM. In HRCT visual assessment, emphysema, tree-in-bud sign, and bronchial wall thickening have higher incidence in SAD defined by each method. (*p* < 0.001). Quantitative CT showed that spirometry-SAD had thicker airway walls (*p* < 0.001), smaller lumens (*p* = 0.011), fewer bronchi (*p* < 0.001), while PRM-SAD had slender blood vessels. Predictive factors for spirometry-SAD were age, male gender, the volume percentage of emphysema in PRM (PRM^Emph^), tree-in-bud sign, bronchial wall thickening, bronchial count; for PRM-SAD were age, male gender, BMI, tree-in-bud sign, emphysema, the percentage of blood vessel volume with a cross-sectional area less than 1 mm^2^ (BV1/TBV). The area under curve (AUC) values for the fitted predictive models were 0.855 and 0.808 respectively.

**Conclusions:**

Compared with PRM, SAD defined by spirometry is more closely related to airway morphology, while PRM is sensitive to early pulmonary dysfunction but may be interfered by pulmonary vessels. Models combining patient information and HRCT assessment have good predictive value for SAD.

**Critical relevance statement:**

HRCT reveals lung structural differences in small airway dysfunction defined by spirometry and parametric response mapping. This insight aids in understanding methodological differences and developing radiological tools for small airways that align with pathophysiology.

**Key Points:**

- Spirometry-SAD shows thickened airway walls, narrowed lumen, and reduced branch count, which are closely related to airway morphology.
- PRM shows good sensitivity to early pulmonary dysfunction, although its assessment of SAD based on gas trapping may be affected by the density of pulmonary vessels and other lung structures.
- Combining patient information and HRCT features, the fitted model has good predictive performance for SAD defined by both spirometry and PRM (AUC values are 0.855 and 0.808, respectively).

**Graphical Abstract:**

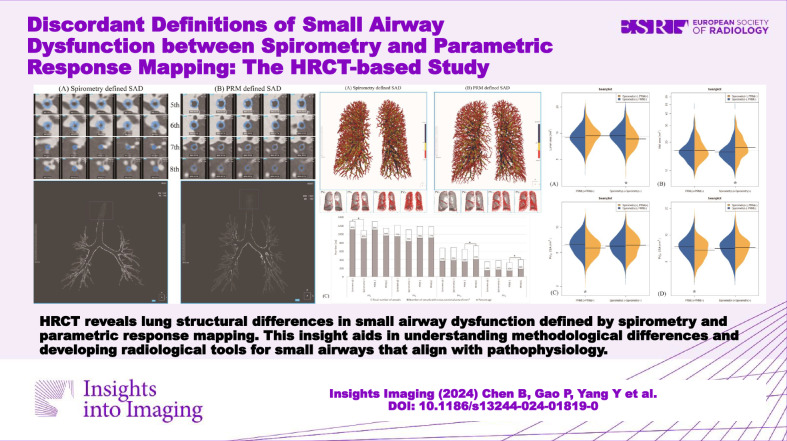

## Introduction

Small airway dysfunction (SAD) is the early pathological basis of respiratory diseases [1–3], with pathological tissues and micro-CT showing abnormalities in the small airways even before symptoms and emphysema appear [1, 2, 4–6]. Reducing irritants such as tobacco smoke and occupational dust exposure can prevent further disease progression. Since the small airways are not the main site of airflow resistance in normal or early pathological conditions, early SAD is difficult to detect, making appropriate evaluation methods crucial for SAD screening.

Methods such as spirometry, forced oscillation technique, and nitrogen washout test can be used for small airway evaluation [2], with spirometry being the most common. It assesses small airway function by measuring the volume of inhaled and exhaled air and the speed of exhalation [7]. However, due to its poor repeatability and lack of a unified gold standard, spirometry is not suitable for epidemiological studies [8], necessitating the exploration of new means for small airway evaluation. In recent years, parameter response mapping (PRM) proposed by Galbán et al [9] has received considerable attention. It is a method based on inspiratory-expiratory CT spatial registration, which locates and quantifies both emphysematous and non-emphysematous (functional small airway disease, fSAD) gas trapping in three-dimensional space through changes in lung gas content in two-phase CT. Many studies have used the volume percentage of fSAD in PRM (PRM^fSAD^) representing non-emphysematous gas trapping as an indicator for evaluating small airways [10–14]. Compared with traditional spirometry, PRM offers the advantages of spatial visualization and numerical repeatability. Unfortunately, no study has reported the difference between spirometry and PRM in small airway evaluation. Although both methods use dynamic breathing process to evaluate lung parenchyma and small airway status, there are still differences in mechanism, which is a question worth exploring.

High-resolution computed tomography (HRCT) can reveal the small structures and subtle changes in the lungs, and with the advancement of computer science and artificial intelligence [15, 16], it can also provide quantitative parameters of lung structure. This is extremely useful for the diagnosis and monitoring of lung structures (especially medium-sized airways and small pulmonary vessels) [13, 17–20]. In this study, we evaluated the lung structure of SAD defined by spirometry and PRM using HRCT, identified the relevant factors, discussed the impact of different mechanisms on SAD assessment, established corresponding prediction models, and proposed ideas for developing new SAD assessment tools.

## Materials and methods

### Study participants

**Fig. 1:**
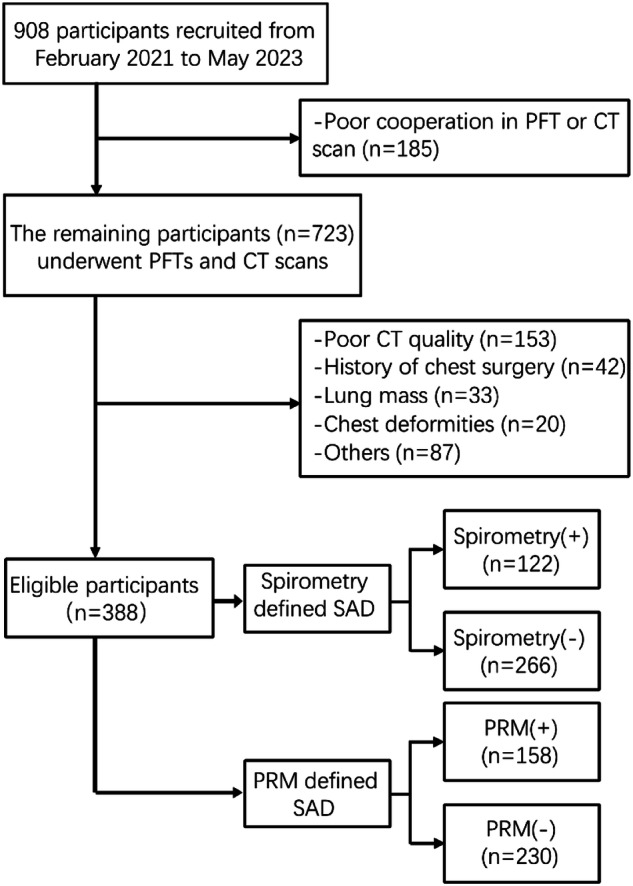
Flowchart of participant inclusion and exclusion. Participants who were unable to complete PFT or CT scans due to poor cooperation were first excluded (*n* = 185). Next, those with poor quality CT images after scanning (*n* = 153), history of chest surgery (*n* = 42), lung mass (*n* = 33), chest deformity (*n* = 20) that affected image analysis were excluded. In addition, participants who were unable to further participate in the study for any other reason were also excluded from the study (*n* = 87), see supplementary materials. The 388 eligible participants were diagnosed with SAD through spirometry and PRM, respectively. Spirometry (+) was defined when at least two of the three indicators (MMEF, FEF 50%, and FEF 75%) were below 65% of the predicted value; otherwise, Spirometry (−). PRM (+) was defined when PRM^fSAD^ was greater than 20%; otherwise, PRM (−). PFT, pulmonary function test; CT, computed tomography; SAD, small airway dysfunction; PRM, parametric response mapping; FVC, forced vital capacity; MMEF, maximum mid expiratory flow at 25–75% of FVC; FEF 50%, forced expiratory flow at 50% of FVC; FEF 75%, forced expiratory flow at 75% of FVC

From February 2021 to May 2023, 908 patients who underwent health check-ups at our hospital and had no acute respiratory symptoms within one month participated in this prospective study. A total of 388 eligible participants were included in the analysis. The study was approved by the Ethics Committee of our Hospital (approval number: 2021K018), and informed consent was obtained from all participants. Participants underwent pulmonary function tests (PFTs), followed by inspiratory and expiratory chest HRCT scans within 2 weeks. The flow chart of participant recruitment is shown in Fig. 1.

### HRCT imaging protocol

The CT imaging protocol was recommended by the Fleischner Society statement [17]. Details of the respiratory training and scanning parameters can be found in the supplementary materials.

### HRCT visual evaluation

We developed a visual assessment protocol for chest HRCT based on the Fleischner Society statement [17]. Any lung region with abnormal low attenuation areas exceeding 5% was considered to have emphysema [21, 22]. Tree-in-bud sign was defined as multiple centrilobular nodules with a linear branching pattern on CT [23, 24], and the presence of this sign in this study was defined as lesions distributed over more than one lung segment or more than three lesions in a single lung segment. Bronchial wall thickening was defined as the thickness of the bronchial wall outside the hilar region being greater than 50% of the diameter of its adjacent pulmonary arteries [25, 26], while bronchiectasis was measured by an airway-artery diameter ratio >1 [27]. Other details can be found in the supplementary materials. CT images were independently evaluated by two radiologists, each with over 5 and 10 years of experience respectively, who were unaware of the clinical information of the participants. Any discrepancies were resolved through discussion.

### Quantitative CT

An automated quantitative analysis of HRCT was performed using lung analysis software (Aview, Coreline Soft, Seoul, Korea). Based on the automatic segmentation of the entire tracheobronchial tree, we extracted airway quantitative parameters at the whole lung and 5th–8th generation levels, including lumen area, wall area, lumen diameter, wall thickness, branch count, etc. We calculated the square root of wall area for airway with internal perimeter of 10 mm (AWT-Pi10) by linear regression, representing standardized bronchial wall thickness [28]. For pulmonary vessels, we segmented them using the software’s prior algorithm, and calculated the blood vessel volumes with different cross-sectional areas. We also obtained the number, diameter, cross-sectional area and surface area of pulmonary vessels at different distances from the pleura. A radiologist with more than 2 years of image processing experience supervised the CT quantitative analysis.

### Parametric response mapping

The principle of PRM is to spatially register inspiratory and expiratory CT scans on a voxel-to-voxel basis, and classify all voxel pairs based on the CT attenuation of Hounsfield units (HU) [9]. Lung voxels with inspiratory attenuation < −950 HU and expiratory attenuation < −856 HU are classified as emphysema, while lung voxels with inspiratory attenuation > −950 HU and expiratory attenuation < −855 HU are classified as functional small airway disease voxels, representing areas of non-emphysematous gas trapping. Referencing previous related studies [10, 11, 13, 14], we used a volume percentage of functional small airway disease in PRM (PRM^fSAD^) > 20% as the definition of small airway dysfunction by PRM (PRM-SAD). PRM analysis in this study was performed using the Aview software.

### Pulmonary function test

Pulmonary function tests for all participants were performed on the MasterScreen PFT System (Jaeger Ltd, Hochberg, Germany) according to the standards of the American Thoracic Society and the European Respiratory Society [29]. Participants were required to perform up to 3 forced expiratory maneuvers, ensuring that forced vital capacity (FVC) and forced expiratory volume in the first second (FEV1) were repeatable within 150 mL, and those who could not cooperate were excluded from the study. As in previous studies [30–34], we used three spirometry indicators to assess small airway dysfunction: maximum mid expiratory flow at 25–75% of FVC (MMEF), forced expiratory flow at 50% of FVC (FEF 50%), and forced expiratory flow at 75% of FVC (FEF 75%). Small airway dysfunction was defined by spirometry (spirometry-SAD) when at least two of these three indicators were below 65% of the predicted value.

### Statistical analysis

Statistical analyses were conducted using SPSS software (Windows version 23.0; Chicago, IL, USA), R software (Windows version 4.3.0), and MedCalc software (Windows version 16.8.4). Participants were dichotomized for comparison based on spirometry and PRM, namely Spirometry(+) vs Spirometry(−) and PRM(+) vs PRM(−); they were also divided into four non-overlapping subgroups by combining both methods: Spirometry(+)PRM(+), Spirometry(+)PRM(−), Spirometry(−)PRM(+), Spirometry(−)PRM(−). Categorical variables were presented as frequencies (percentages) and analyzed using chi-square tests or Fisher’s exact test. Continuous variables were presented as mean ± standard deviation (SD) and analyzed using one-way analysis of variance (ANOVA), with least significant difference (LSD) employed for intergroup comparisons; Welch’s ANOVA, Brown-Forsythe ANOVA, or non-parametric tests were utilized in cases of unequal variances. Clinical data and imaging variables, after collinearity diagnosis and manual exclusion of variables with a variance inflation factor (VIF) > 5 or tolerance < 0.2, were entered into a logistic regression model using a forward conditional stepwise method to determine predictive factors for SAD. Statistical significance was set at a two-sided *p* value of less than 0.05.

## Results

### Participant characteristics

As shown in Fig. 1, 388 participants (229 males, 159 females) with an average age of 63.5 years met the inclusion criteria. SAD was diagnosed in 31.4% of participants by spirometry and in 40.7% by PRM, as detailed in Supplementary Table E1.

Table 1 outlines the characteristics of four subgroups defined by both diagnostic methods: Spirometry(+)PRM(+), Spirometry(+)PRM(−), Spirometry(−)PRM(+), and Spirometry(−)PRM(−), referred to as Groups A, B, C, and D, respectively. It also contrasts the characteristics between the positive and negative groups defined by each method, Spirometry(+) vs Spirometry(−) and PRM(+) vs PRM(−), with specific features and values contained in Supplementary Table E2.

**Table 1:** Demographics, spirometry and HRCT assessments of participants

	Spirometry (+) PRM (+) (*n* = 75)	Spirometry (+) PRM (−) (*n* = 47)	Spirometry (−) PRM (+) (*n* = 83)	Spirometry (−) PRM (−) (*n* = 183)	Spirometry (+) vs (−)	PRM (+) vs (−)
Group	A	B	C	D	(A + B) vs (C + D)	(A + C) vs (B + D)
Clinical data						
Male sex, *n* (%)	63 (84)	28 (59.6)*	58 (69.9)	80 (43.7)*^	< 0.001^a^	< 0.001^a^
Age, yr	69.5 ± 8.1	63.5 ± 10*	61.5 ± 10.5*	66.3 ± 9.2*	< 0.001^a^	< 0.001^a^
Smoking history, *n* (%)	50 (66.7)	13 (27.7)*	30 (36.1)*	44 (24)*	< 0.001^a^	< 0.001^a^
BMI, kg/m^2^	22.7 ± 3.6	23.8 ± 2.8	22.9 ± 3.3	24.5 ± 3.2*^	0.020	< 0.001
Spirometry						
FVC, %pred	73.9 ± 19.9	85.7 ± 16.6*	96.8 ± 18.7*^#^	93.9 ± 14.9*^#^	< 0.001	0.001
FEV1, %pred	60.3 ± 20.5	80.1 ± 15.3*	109.8 ± 21.8*^#^	104.8 ± 14.6*^#^	< 0.001	< 0.001
FEV1/FVC, %	63.7 ± 12.9	75.1 ± 9.8*	90.6 ± 7*^#^	89.9 ± 6.7*^#^	< 0.001	< 0.001
PRM						
PRM^Emph^ (%)	15.8 ± 12	4.7 ± 8*	6.1 ± 3.9*	1.8 ± 2.6*^	< 0.001^a^	< 0.001^a^
PRM^fSAD^ (%)	39.2 ± 14	10 ± 5.6*	36.7 ± 13.4^#^	8.1 ± 5.5*^	< 0.001^a^	< 0.001^a^
PRM^Normal^ (%)	43.3 ± 19.1	81.8 ± 12.8*	55.4 ± 14.6*^#^	87.2 ± 8.2*^	< 0.001	< 0.001
CT visual evaluation						
Emphysema, *n* (%)	49 (65.3)	7 (14.9)*	24 (28.9)*	4 (2.2)*^#^^	< 0.001^a^	< 0.001^a^
Tree-in-bud sign, *n* (%)	35 (46.7)	11 (23.4)	26 (31.3)	18 (9.8)*^	< 0.001^a^	< 0.001^a^
BWT, *n* (%)	34 (43.5)	10 (21.3)*	3 (3.6)*^#^	7 (3.8)*^#^	< 0.001^a^	< 0.001^a^
Bronchial dilation, *n* (%)	17 (22.7)	11 (23.4)	29 (34.9)	53 (29)	0.110	0.782
CT quantitative evaluation						
AWT-Pi10	3.9 ± 0.8	3.8 ± 0.8	3.1 ± 0.7*^#^	3.4 ± 0.8*^#^^	< 0.001^a^	0.686
Branch count, ea	239.3 ± 101.1	222.9 ± 88.5	304.5 ± 86.4*^#^	268.3 ± 76.2*^#^^	< 0.001	0.108
Lumen area, mm^2^	9.1 ± 2.6	8.6 ± 2.4	10.1 ± 2.6*^#^	9.4 ± 2.5^#^^	0.011	0.108
Wall area, mm^2^	18.8 ± 4.8	17.6 ± 4.8	15.3 ± 3.5*^#^	16.6 ± 4.7*	< 0.001^a^	0.837
Wall area, %	60.5 ± 9.7	59.6 ± 10	50.4 ± 8.6*^#^	54.3 ± 9*^#^^	< 0.001^a^	0.806
BV1/TBV	0.03 ± 0.01	0.04 ± 0.01*	0.04 ± 0.01*	0.04 ± 0.01*	< 0.001	0.002
PV_6_ CSA, mm^2^	3.7 ± 1.5	3.5 ± 1.1	3 ± 1*	3.4 ± 1.3^	0.008^a^	0.636
PV_9_ CSA, mm^2^	4.3 ± 1.4	4.3 ± 1.2	3.7 ± 1.1*^#^	4.2 ± 1.4^	0.085	0.054
PV_15_ CSA, mm^2^	6.2 ± 1.8	6.7 ± 1.8	5.6 ± 1.6*^#^	6.5 ± 1.7^	0.359	< 0.001
PV_21_ CSA, mm^2^	7.3 ± 2.1	7.9 ± 2.2	6.7 ± 1.9^#^	7.6 ± 1.9^	0.320	0.003

Data are mean ± SD unless indicated otherwise

*HRCT* high-resolution computed tomography, *PRM* parametric response mapping, *BMI* body mass index, *FVC* forced vital capacity, *FEV1* forced expiratory volume in the first second, *PRM*^*Emph*^ the volume percentage of emphysema in PRM, *PRM*^*fSAD*^ the volume percentage of functional small airway disease in PRM, *PRM*^*Normal*^ the volume percentage of normal area in PRM, *BWT* bronchial wall thickening, *AWT* airway wall thickness, *Pi10* square root of wall area for airway w**i**th internal perimeter of 10 mm, *BV1* blood volume with cross-sectional area less than 1 mm^2^, *TBV* total blood volume, *PV*_*n*_ pulmonary vessel within n mm of the pleura, *CSA* cross-sectional areas

* *p* < 0.05, compared to Spirometry (+) PRM (+); ^#^ *p* < 0.05, compared to Spirometry (+) PRM (−); ^ *p* < 0.05, compared to Spirometry (−) PRM (+)

^a^ The numerical values or percentage frequencies of characteristics in the SAD-positive group are significantly larger

In SAD defined by each method, the proportion of males was significantly higher than in non-SAD (*p* < 0.001), with a higher smoking rate (*p* < 0.001), especially in the Spirometry(+)PRM(+) (Table 1). Additionally, the smoking rate among males (53.3%) was significantly higher than among females (9.4%) (Supplementary Fig. E1). Participants with SAD defined by either method were significantly older (*p* < 0.001), with the highest average age in the Spirometry(+)PRM(+) (*p* < 0.05). The BMI of the PRM-positive subgroup was lower (Table 1).

### Spirometry assessment

**Fig. 2:**
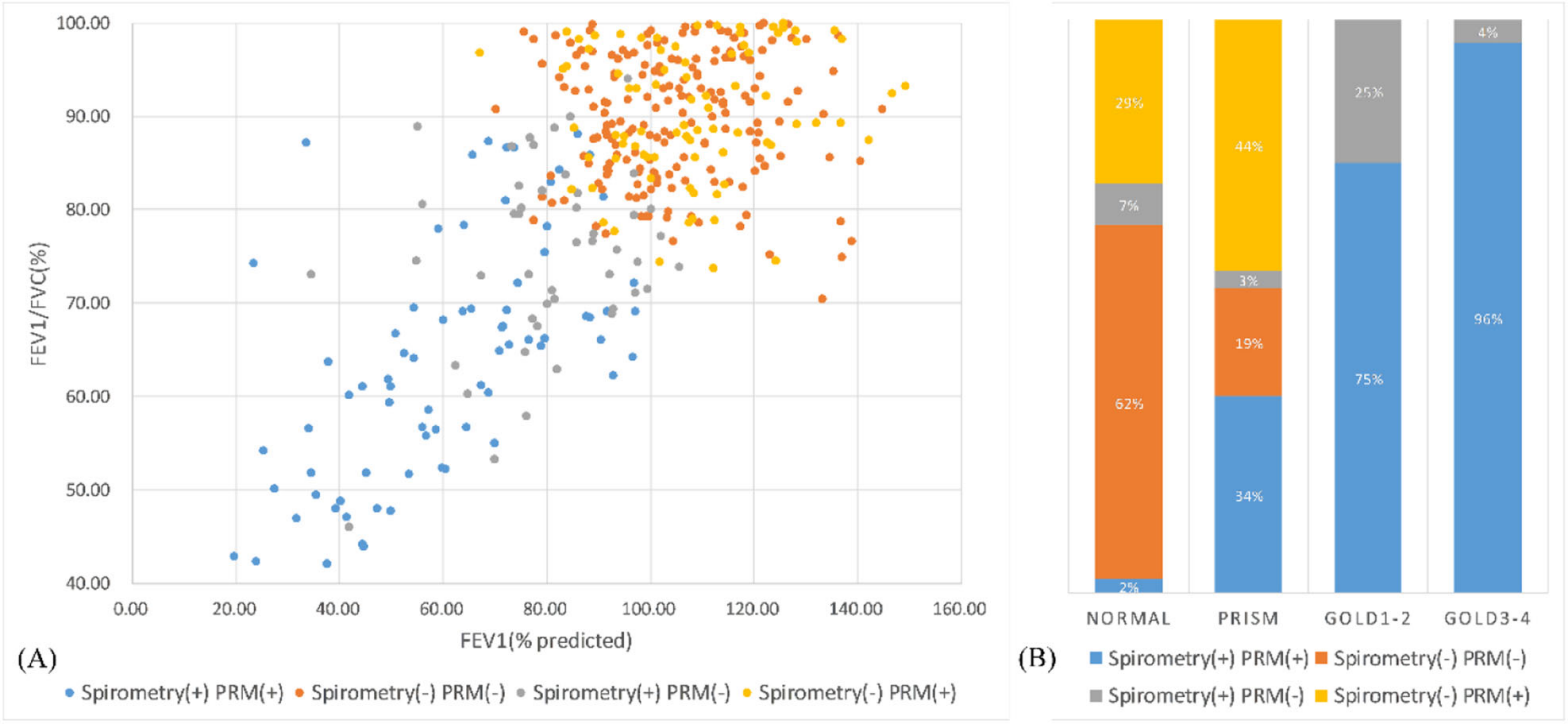
**A** and **B** show the airflow limitation status of SAD defined by two methods and their distribution and overlap in different GOLD stages. Spirometry (+) was defined when at least two of the three indicators (MMEF, FEF 50%, and FEF 75%) were below 65% of the predicted value; otherwise, Spirometry (−). PRM (+) was defined when PRM^fSAD^ was greater than 20%; otherwise, PRM, (−). SAD, small airway dysfunction; GOLD, global initiative for chronic obstructive lung disease; PRM, parametric response mapping; FEV1, forced expiratory volume in 1 s; FVC, forced vital capacity; MMEF, maximum mid expiratory flow at 25–75% of FVC; FEF 50%, forced expiratory flow at 50% of FVC; FEF 75%, forced expiratory flow at 75% of FVC

Spirometry indices such as FVC, FEV1, FEV1/FVC, FEF50%, FEF75%, and MMEF were significantly lower in PRM-SAD (*p* < 0.05), with Spirometry(+)PRM(+) showing the lowest values, followed by Spirometry(−)PRM(+) (Table 1, Supplementary Tables E2–E4). According to the Global Initiative for Chronic Obstructive Lung Disease (GOLD) grading criteria, there was a considerable overlap between spirometry and PRM in defining SAD across the normal (64%), PRISm (53%), GOLD 1–2 (75%), and GOLD 3–4 (96%) groups (Fig. 2, Supplementary Table E5).

### HRCT assessment

In PRM, PRM^fSAD^ and PRM^Emph^ were also significantly higher in spirometry-SAD (*p* < 0.001), with Spirometry(+)PRM(+) having the largest volumes, followed by Spirometry(−)PRM(+) (Table 1, Supplementary Table E2).

CT visual assessment (Table 1, Supplementary Tables E2, E6 and E7) indicated that emphysema, tree-in-bud sign, and bronchial wall thickening were significantly more prevalent in SAD defined by each of the two methods (*p* < 0.001), with the highest frequency in Spirometry(+)PRM(+).

**Fig. 3:**
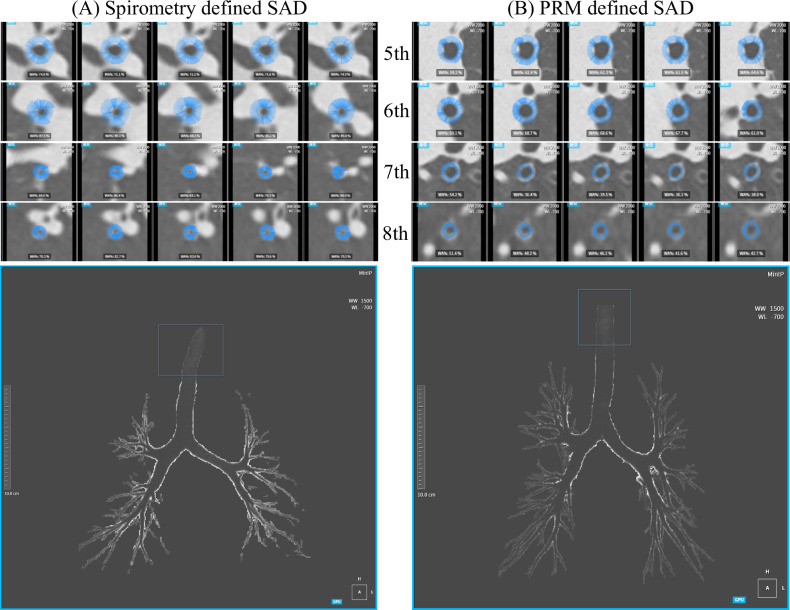
The upper part shows the cross-sections of the 5th to 8th generation bronchi as displayed by HRCT, with the blue rings representing the bronchial walls. The lower part shows the bronchial tree visualized by using the MinIP algorithm. **A** Shows a 67-year-old female participant with spirometry-SAD, with the average WA% of the 5th to 8th generations being 79.2%, 79.6%, 74.7%, and 69.1% respectively; **B** shows a 57-year-old female participant with PRM-SAD, with the average WA% of the 5th to 8th generations being 43.5%, 40.2%, 46.7%, and 43.1% respectively. SAD, small airway dysfunction; PRM, parametric response mapping; MinIP, minimum intensity projection; WA%, wall area percentage

In HRCT quantitative analysis, spirometry-SAD showed greater airway wall thickness (AWT-Pi10), wall area, and wall area percentage (WA% = WA/(WA + LA) × 100%) with smaller lumen and fewer branches (*p* < 0.05) at the whole lung level compared to non-spirometry-SAD (Table 1, Supplementary Table E2, Supplementary Fig. E2). Similar patterns were observed at the 5th–8th generation airway level (Fig. 3, Supplementary Table E11). Conversely, PRM-SAD showed mostly no significant difference in airway quantitative parameters at the whole lung level compared to non-PRM-SAD (Table 1, Supplementary Table E2, Supplementary Fig. E2), but had fewer branch count at the 5th–7th generation level (*p* < 0.05) and larger lumen at the 6th–8th generation level (*p* < 0.05) (Supplementary Table E11, Fig. 3); AWT-Pi10 and WA% showed no significant difference (Supplementary Table E11). Among subgroups, at the whole lung level, Spirometry(+)PRM(+) and Spirometry(+)PRM(−) had larger AWT-Pi10 and WA%, smaller lumen, and fewer branches, while Spirometry(−)PRM(+) had the smallest AWT-Pi10 and largest lumen area (Table 1). At the 6th–8th generation level, Spirometry(+)PRM(−) had the smallest lumen, with other parameters similar to the whole lung level (Supplementary Table E10).

**Fig. 4:**
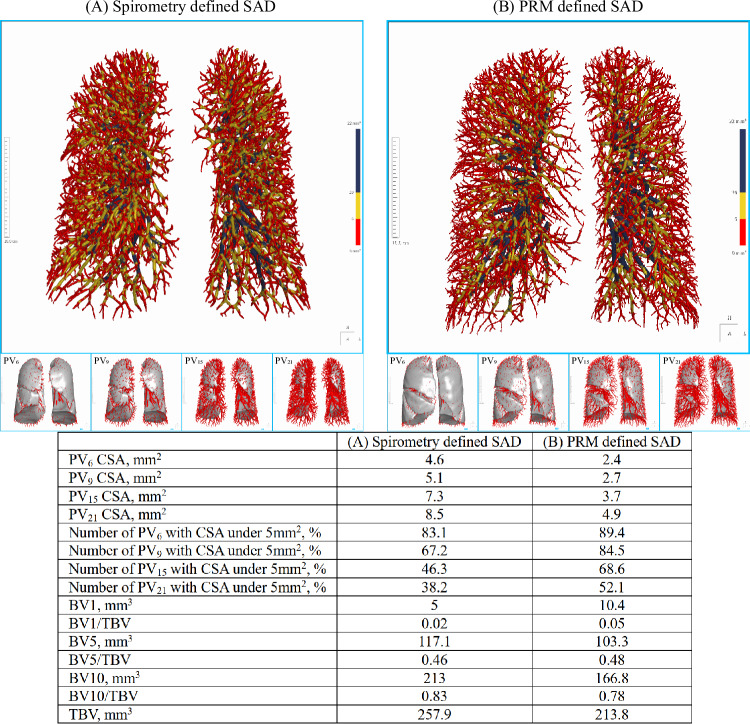
**A** and **B** show the pulmonary vascular trees of SAD by spirometry and PRM, respectively, with vessels at different distances from the pleura below. The vessel colors indicate CSA ranges: red for CSA < 5 mm^2^, yellow for 5 mm^2^ ≤ CSA < 10 mm^2^, and blue for 10 mm^2^ ≤ CSA < 22 mm^2^. **A** represents a 64-year-old male participant belonging to the Spirometry(+)PRM(−) subgroup, and **B** represents a 57-year-old female participant belonging to the Spirometry(−)PRM(+) subgroup. The specific pulmonary vascular parameters for both are displayed in the table below the images. SAD, small airway dysfunction; PRM, parametric response mapping; CSA, cross-sectional areas; PV_n_, pulmonary vessel within n mm of the pleura; BV1, blood volume with cross-sectional area less than 1 mm^2^; BV5, blood volume with cross-sectional area less than 5 mm^2^; BV10, blood volume with cross-sectional area less than 10 mm^2^; TBV, total blood volume

Total blood volume (TBV) in the lung was larger in SAD as defined by each method (Supplementary Table E13), with similar patterns across subgroups, especially in Spirometry(+)PRM(+) (*p* < 0.001) (Supplementary Table E12). Spirometry-SAD exhibited a larger vascular cross-sectional area (CSA) and diameter, especially within 6 mm of the pleura (*p* < 0.05), while PRM-SAD had a smaller CSA and diameter, and a higher proportion of small vessels (CSA < 5 mm^2^) within 15 mm and 21 mm of the pleura (*p* < 0.05) (Table 1, Supplementary Table E15, Fig. 4). Among subgroups, Spirometry(−)PRM(+) generally had smaller CSA at distances of 6, 9, 15, and 21 mm from the pleura, with a higher percentage of small vessels (*p* < 0.05) (Supplementary Table E14).

### Binary logistic regression analysis

Clinical data and HRCT evaluation indices (Supplementary Tables E2, E4, E7, E9, E11, E13 and E15) were analyzed using binary logistic regression (*p* < 0.05). The results showed that age, male gender, PRM^Emph^, tree-in-bud sign, bronchial wall thickening, and airway branch count were predictive factors for spirometry-SAD (Table 2), with a fitted model AUC of 0.855 (95% CI: 0.809–0.902) and 84% prediction accuracy (Supplementary Table E16). For PRM-SAD, age, male gender, BMI, tree-in-bud sign, emphysema, and BV1/TBV were predictive factors (Table 3), with a fitted model AUC of 0.808 (95% CI: 0.763–0.852) and 76.8% prediction accuracy (Supplementary Table E17). Both models had Hosmer-Lemeshow test *p* values > 0.05, indicating good fit.

**Table 2:** Variables in logistic regression analysis for predicting SAD defined by spirometry

	OR (95% CI)	*p* value
Age	1.047 (1.016 – 1.079)	0.003
Male	2.032 (1.056 – 3.908)	0.034
PRM^Emph^	1.140 (1.083 – 1.200)	< 0.001
Tree-in-bud sign	2.051 (1.038 – 4.052)	0.039
BWT	6.394 (2.730 – 14.973)	< 0.001
Branch count	0.990 (0.986 – 0.993)	< 0.001

*SAD* small airway dysfunction, *OR* odds ratio, *CI* confidence interval, *BWT* bronchial wall thickening, *PRM* parametric response mapping, *PRM*^*Emph*^ the volume percentage of emphysema in PRM

**Table 3:** Variables in logistic regression analysis for predicting SAD defined by PRM

	OR (95% CI)	*p* value
Age	1.028 (1.003 – 1.054)	0.030
Male	1.908 (1.131 – 3.218)	0.015
BMI	0.878 (0.809 – 0.953)	0.002
Tree-in-bud sign	2.130 (1.151 – 3.944)	0.016
Emphysema	8.151 (3.880 – 17.120)	< 0.001
BV1/TBV	1.67 × 10^−12^ (6.43 × 10^−21^ – 4.33 × 10^−4^)	0.006

*SAD* small airway dysfunction, *PRM* parametric response mapping, *OR* odds ratio, *CI* confidence interval, *BMI* body mass index, *BV1* blood volume with cross-sectional area less than 1 mm^2^, *TBV* total blood volume

## Discussion

We first compared spirometry and PRM, two different methods of assessing small airways. The participants with SAD defined by both methods had similar clinical characteristics and a good overlap in different degrees of airflow limitation, but differed in airways and pulmonary vessels on HRCT. Our goal was to analyze predictive factors associated with the two diagnostic methods, explore the mechanistic differences between them, and provide insights for methodological advancements in SAD prediction.

A large cross-sectional study in China showed that the prevalence of SAD by spirometry was 43.5%, with older age, female gender, smoking, high BMI, and respiratory symptoms (including a history of chronic cough, childhood pneumonia or bronchitis) being risk factors [30]. In our cohort, the prevalence of SAD by spirometry was 31.4%, and by PRM was 40.7%. Similar to their findings, older age was also an independent risk factor in our study, aligning with the physiological loss of lung elastic recoil and terminal bronchioles associated with aging, which affects spirometry indicators [35–37]. Male gender was an independent risk factor for SAD in our research, and the BMI was lower in the SAD group. This could be attributed to the higher proportion of participants under 50 years of age in their study (48.2%) compared to ours (10%). In other related studies, SAD in younger populations was more likely associated with asthma, while high BMI was identified as a risk factor for asthma. Additionally, male gender and low BMI were found to be risk factors for COPD [38–40]. After multivariate adjustment, low BMI remained an independent risk factor for PRM-SAD in our study, which does not exclude the possibility of image noise alteration mediated by BMI, warranting further investigation.

The small airways, often referred to as the silent zone of the lung, can develop functional impairments that are not easily detected. These impairments may occur due to environmental stimuli such as tobacco smoke, occupational dust exposure, and environmental pollution [38–40]. This impairment can gradually progress to irreversible obstructive lung diseases through various mechanisms [41–43] such as airway loss, wall thickening, mucus obstruction or alveolar attachment loss [1, 2, 4, 44]. Medium-sized airways are an extension of the small airways and can largely reflect the status of the small airways. In this study, HRCT showed that spirometry-SAD had reduced branch count, increased wall thickness and narrowed lumen, both at the whole lung level and the middle airway level (5–8 generations), consistent with the results of Lu et al [20]. Furthermore, branch count and visually assessed tree-in-bud sign and bronchial wall thickening on HRCT were independently associated with spirometry-SAD after multivariate adjustment, indicating a close relationship between spirometry and airway structure.

PRM-SAD had no obvious changes in airway structure at the whole lung level. However, subgroup analysis revealed that the Spirometry(−)PRM(+) showed the smallest WA% and the largest lumen, both at the whole lung level and at the 5th to 8th generation airway level. This finding fills the research gap between PRM and quantitative CT morphological relationships in medium-sized airways. Combined with the tree-in-bud sign as an independent risk factor, it suggests that PRM-SAD may be related to mucus obstruction and lumen dilatation, likely mediated by gas trapping. This contrasts with the observations of Vasilescu et al [12], who noted through micro-CT that PRM^fSAD^ was associated with terminal bronchiole loss, lumen narrowing, and wall thickening. The possible reasons for this discrepancy might be that our study only measured airways at the intermediate level, and another reason could be that their study subjects were few and all had end-stage COPD.

Alveolar attachments are connective tissue septa linking airways and alveoli. Their loss reduces radial traction, causing premature airway closure and gas trapping during exhalation [44]. This early pathological change is observable even in pre-COPD patients without spirometry-detected airflow obstruction [5]. In quantitative CT, we analyzed medium-sized airway indicators on inspiratory CT, where the pressure difference inside and outside the airways increased. Early functional impairment shows that while the airway wall hasn’t significantly thickened, alveolar attachments are lost, yet the airway remains elastic, allowing significant lumen expansion during inspiration. This may relate to PRM-SAD morphological changes, such as reduced airway wall area and increased luminal expansion. Thus, PRM-SAD might indicate early alveolar attachment loss, warranting further research. Previous studies support PRM’s relation to alveolar attachments [12]. Additionally, PRM^Emph^ is an independent correlate of spirometry-SAD in this study, consistent with other research [4, 45], suggesting SAD may precede emphysema. PRISm, a preclinical COPD state, was defined as SAD in 81% of cases in this study, with over half identified solely by PRM (Fig. 2). This demonstrates PRM’s sensitivity in assessing early small airway function. Further analysis of the correlation between lung histopathology in smokers or early obstructive pulmonary disease patients and PRM could yield more comprehensive conclusions.

Gas trapping impairs lung tissue gas exchange, causing local hypoxia, vascular proliferation, and wall thickening, while lung parenchymal destruction reduces the number of small pulmonary vessels [18, 46, 47]. In this study, HRCT showed that spirometry-SAD had thicker pulmonary vessels, possibly due to vascular smooth muscle hypertrophy in vascular remodeling [47]. However, PRM-SAD is characterized by thinner pulmonary vessels and a higher proportion of small vessels (CSA < 5 mm^2^), similar to Ritchie et al ’s findings [6], which showed a higher ratio of small pulmonary vessels in smokers compared to non-smokers, correlating with an accelerated decline in FEV1. This phenomenon might be due to more small vessels entering the scanner’s resolution range or gas trapping causing vascular constriction [48]. Additionally, thinner vessels and reduced blood perfusion could lower lung density, similar to the mosaic perfusion pattern seen on CT, potentially interfering with PRM’s gas trapping assessment. Although we analyzed lung parenchyma between −500 HU and −1000 HU to minimize the impact of airways and vessels on density [9], this measure could not completely avoid interference. Further research is needed to verify the cause-and-effect relationship between gas trapping and thinner pulmonary vessels. Additionally, BV1/TBV was a beneficial factor in the logistic regression model, indicating that a lower percentage of small vessel volume (CSA < 1 mm^2^) is more likely associated with PRM-SAD. This seems contradictory to the number of vessels with a CSA < 5 mm^2^, but it may relate to the lengths, numbers, and morphology of small pulmonary vessels beyond 21 mm from the pleura. Due to the complexity, further data and statistical analysis are required for interpretation.

Although PRM may be affected by the density of other lung structures due to its mechanism, various studies have shown that PRM^fSAD^ correlates well with lung function indicators such as FEV1, FEV1/FVC, total lung capacity, alveolar volume, and residual volume [9, 10, 20]. In this study, it also overlaps significantly with spirometry-defined SAD at different GOLD stages and is more sensitive than spirometry in PRISm, thus reflecting small airway function to some extent. Future methods may better eliminate density interference from pulmonary vessels, airways, and lung interstitium. Based on this, combined with airway structure information, a three-dimensional spatial CT method conforming to pathophysiology could be developed for small airway assessment.

Our study also has some limitations. First, the attenuation value of voxels in PRM is affected by various factors. We have unified the CT scan parameters and reconstruction kernel, but the lack of respiratory gating may cause errors in gas trapping analysis [19]. In addition, our hospital is a comprehensive hospital with elderly characteristics, so the patients are generally older. We are planning to conduct a multi-center study to increase the sample size and diversity. Finally, the results lack histological evidence, because obtaining tissue samples is difficult, especially for early obstructive lung diseases.

## Conclusion

Spirometry is closely related to airway morphology, and PRM has a high sensitivity in identifying early SAD. Since PRM identifies gas trapping through lung parenchymal density, it may be influenced by other lung structures, which could reduce its correlation with airway morphology. Nevertheless, PRM remains an effective method for assessing small airway function. These insights pave the way for the development of improved tools for SAD assessment.

## Supplementary information

ELECTRONIC SUPPLEMENTARY MATERIAL

## Data Availability

All the data will be shared upon reasonable request by the corresponding author.

## Abbreviations

AUC: Area under the ROC curve
AWT: Airway wall thickness
BMI: Body mass index
BV1: Blood volume with cross-sectional area less than 1 mm^2^
BWT: Bronchial wall thickening
CSA: Cross-sectional areas
FEF 50%: Forced expiratory flow at 50% of FVC
FEF 75%: Forced expiratory flow at 75% of FVC
FEV1: Forced expiratory volume in the first second
FVC: Forced vital capacity
GOLD: Global initiative for chronic obstructive lung disease
HRCT: High-resolution computed tomography
MMEF: Maximum mid expiratory flow at 25–75% of FVC
PFT: Pulmonary function test
Pi10: Square root of wall area for airway with internal perimeter of 10 mm
PRISm: Preserved ratio and impaired spirometry
PRM: Parametric response mapping
PRM^Emph^: The volume percentage of emphysema in PRM
PRM^fSAD^: The volume percentage of functional small airway disease in PRM
PV_n_: Pulmonary vessel within n mm of the pleura
ROC: Receiver operating characteristic
SAD: Small airway dysfunction
TBV: Total blood volume
