# Supplementary material for: Discordant definitions of small airway dysfunction between spirometry and parametric response mapping: the HRCT-based study
Source: Insights Imaging. 2024 Oct 2;15:233. doi: 10.1186/s13244-024-01819-0 (PMC11447176; doi:10.1186/s13244-024-01819-0)
Supplement: Supplementary file 1 — ELECTRONIC SUPPLEMENTARY MATERIAL [file 13244_2024_1819_MOESM1_ESM.pdf]

Discordant Definitions of Small Airway Dysfunction between Spirometry and Parametric Response Mapping: The HRCT-based Study

ELECTRONIC SUPPLEMENTARY MATERIAL

Table of Contents

Supplementary Information on Inclusion and Exclusion Criteria.................................2
HRCT Imaging Protocol 2
Supplementary Information on HRCT Visual Evaluation 3
Supplementary Information on Quantitative CT 3
Supplementary Figures 4
Supplementary Tables...7
Basic Characteristics..7
Comparison of Two Methods Grouping...8

Supplementary Information on Inclusion and Exclusion Criteria

CT image quality was assessed based on the presence of artifacts (including motion and metal artifacts), completeness of both lung structures, and inflation status of expiratory or inspiratory phase [1]. The trachea is often used as a reference for assessing the adequacy of respiratory gas: during full inspiration, the trachea is round/oval, and during full expiration, the posterior membranous wall of the trachea bends, making the trachea crescent-shaped [2]. Any clear, discrete or approximately circular pulmonary lesion with a diameter greater than 3 cm was defined as a lung mass [3; 4]. Chest deformities refer to structural abnormalities of the chest wall, and this study excluded obvious pectus excavatum and pectus carinatum that could affect lung analysis [5]. Others included missing data (any of demographic characteristics, CT images, PFT data), pulmonary inflammation (although people with acute respiratory symptoms were excluded in advance, some participants still showed inflammatory exudation on CT images), significant pleural effusion, multiple old tuberculous lesions, and isolated lung.

HRCT Imaging Protocol

Before the CT scan, the participants underwent multiple respiratory trainings, in the order of deep inspiration - breath hold - natural respiration - deep expiration - breath hold - natural respiration to ensure the smooth acquisition of images. In the supine position, a dual-source CT system (Somatom Definition Flash, Siemens Healthcare, Forchheim, Germany) was used to perform chest scans in both full inspiration and expiration states of the participants. The scanning parameters were obtained from the Fleischner Society statement[6], with a collimation of ≤1, a pitch of 1, 120 kVP, and 40-200 mAs for CT scans; using B30f kernel, reconstruction was performed from the thoracic inlet to the lung base with a slice thickness of 1mm.

Supplementary Information on HRCT Visual Evaluation

The emphysema index (EI) refers to the percentage of low-density areas (less than -950 HU) on CT images. Generally, it is believed that when the EI is greater than 5%, it can be diagnosed as emphysema [7; 8]. Tree-in-bud sign indicates that the lesion involves the small airways below the bronchioles. Due to the centrilobular dilation of the airways and the filling of the lumen with mucus, pus, and other inflammatory substances, it appears on thin-slice CT or HRCT as centrilobular soft tissue density nodules with a diameter of 2-4 mm and branching linear shadows connected to them. This sign, resembling the budding of tree branches in spring, is a common inflammatory sign in the lungs [9; 10]. Most studies compare the diameter of adjacent pulmonary arteries to assess bronchial wall thickening (BWT). According to the thickness of the bronchial wall compared to the diameter of the adjacent pulmonary artery, it can be graded as: Grade 1, less than 50% of the artery diameter; Grade 2, 50-100% of the artery diameter; Grade 3, greater than 100% of the artery diameter [11; 12]. Therefore, this study uses a bronchial wall thickness greater than 50% of the adjacent pulmonary artery diameter as the criterion for judging bronchial wall thickening (BWT).

Supplementary Information on Quantitative CT

An automated quantitative analysis of lung HRCT was performed using the software Aview (Coreline Soft, Seoul, Korea).

Detailed information can be found at https://www.corelinesoft.com/en/solutions/copd and https://grand-challenge.org/aiforradiology/product/coreline-soft-aview-copd/.

References:

1 Regan EA, Hokanson JE, Murphy JR et al (2010) Genetic Epidemiology of COPD (COPDGene) Study Design. Copd: Journal of Chronic Obstructive Pulmonary Disease 7:32-43

2 Mudgal P, Murphy A, Lorente E (2023) HRCT chest - expiration (protocol). Available via https://doi.org/10.53347/rID-28654 (Accessed on 18 Jan 2024)

3 Jones J, Bell D, Tatco V (2022) Pulmonary mass. Radiopaedia.org. Available via https://doi.org/10.53347/rID-14509 (Accessed on 18 Jan 2024)

4 Webb WR, Higgins CB. (2016) Thoracic Imaging: Pulmonary and Cardiovascular Radiology: Wolters Kluwer Health

5 Cobben JM, Oostra RJ, van Dijk FS (2014) Pectus excavatum and carinatum. European Journal of Medical Genetics 57:414-417
6 Lynch DA, Austin JH, Hogg JC et al (2015) CT-Definable Subtypes of Chronic Obstructive Pulmonary Disease: A Statement of the Fleischner Society. Radiology 277:192-205
7 Park J, Hobbs BD, Crapo JD et al (2020) Subtyping COPD by Using Visual and Quantitative CT Imaging Features. Chest 157:47-60
8 Hurst JR, Vestbo J, Anzueto A et al (2010) Susceptibility to exacerbation in chronic obstructive pulmonary disease. The New England Journal of Medicine 363:1128-1138
9 Hansell DM (2001) Small airways diseases: detection and insights with computed tomography. The European Respiratory Journal 17:1294-1313
10 Buckley C, Tucker D, Thorne N, Sieker H (1965) Bronchiolectasis: The clinical syndrome and its relationship to chronic lung disease. The American Journal of Medicine 38:190-198
11 Roberts HR, Wells AU, Milne DG et al (2000) Airflow obstruction in bronchiectasis: correlation between computed tomography features and pulmonary function tests. Thorax 55:198-204
12 Sheehan RE, Wells AU, Copley SJ et al (2002) A comparison of serial computed tomography and functional change in bronchiectasis. European Respiratory Journal 20:581-587

Supplementary Figures

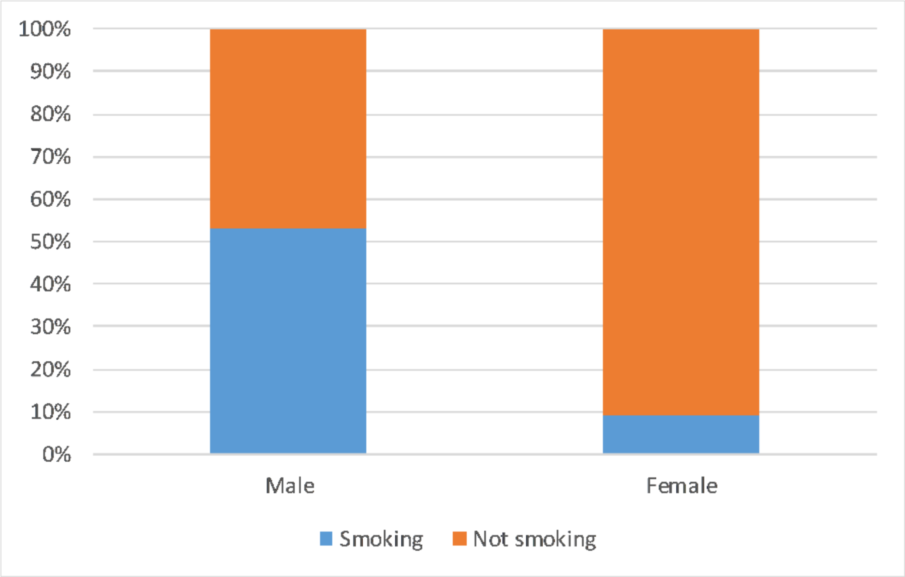

Figure E1: Smoking rates among male and female.

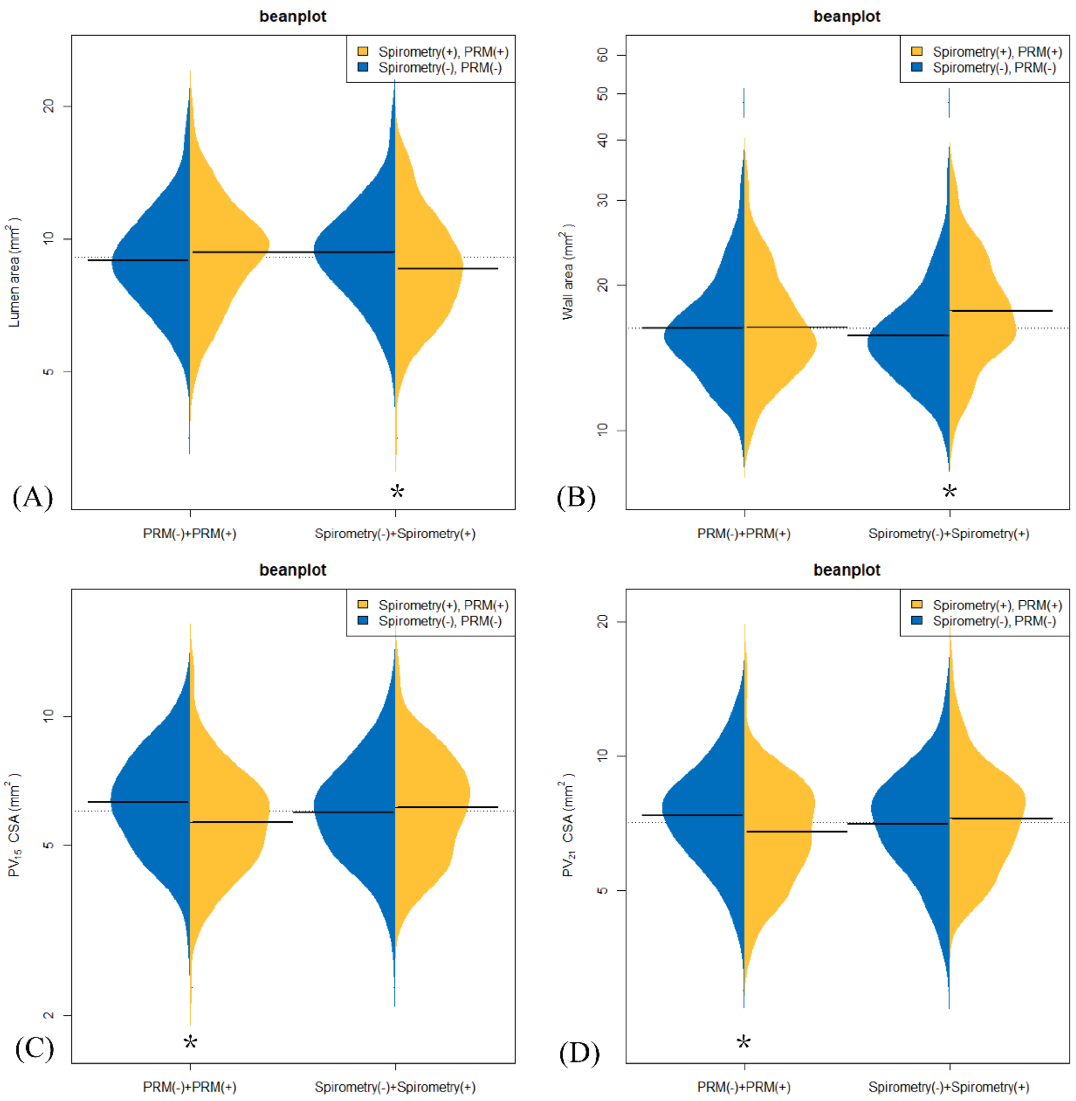

Figure E2. Beanplot of HRCT quantitative parameters. (A) and (B) show the airway lumen area and wall area at the whole lung level, respectively. (C) and (D) show the mean pulmonary vessel cross-sectional area at 15 mm and 21 mm from the pleura, respectively. The yellow part of the beanplot represents SAD defined by the method, and the blue part represents non-SAD. Spirometry (+) was defined when at least two of the three indicators (MMEF, FEF 50%, and FEF 75%) were below 65% of the predicted value; otherwise, Spirometry (-). PRM (+) was defined when PRM^{fSAD} was greater than 20%; otherwise, PRM (-). SAD = small airway dysfunction; PRM = parametric response mapping; CSA = cross-sectional areas; PV_n = pulmonary vessel at n mm from pleura; FVC = forced vital capacity; MMEF = maximum mid expiratory flow at 25-75% of FVC; FEF 50% = forced expiratory flow at 50% of FVC; FEF 75% = forced expiratory flow at 75% of FVC.

Supplementary Tables

Basic Characteristics

Supplementary Table E1: Characteristics of Enrolled Participants

No. of subjects	388
Male sex, n (%)	229 (59)
Age, y	63.5 ± 10.2
Smoking history, n (%)	137 (35.3)
BMI, kg/m^2	23.7 ± 3.3
PRISm, n (%)	32 (8.3)
GOLD 1-2, n (%)	44 (11.3)
GOLD 3-4, n (%)	25 (6.4)
Spirometry defined SAD, n (%)	122 (31.4)
PRM defined SAD, n (%)	158 (40.7)

Note.—Data are mean ± SD unless indicated otherwise. BMI = body mass index, PRISm = preserved ratio and impaired spirometry, GOLD = global initiative for chronic obstructive lung disease, PRM = parametric response mapping, SAD = small airway dysfunction.

Comparison of Two Methods Grouping

Table E2: Demographics, Spirometry and HRCT Assessments of Participants

	Spirometry (-) (n=266)	Spirometry (+) (n=122)	*P* Value	PRM (-) (n=230)	PRM (+) (n=158)	*P* Value
**Clinical data**						
Male sex, n (%)	138 (51.9)	91 (74.6)	< 0.001	108 (47)	121 (76.6)	< 0.001
Age, yr	61.8 ± 10.2	67.2 ± 9.3	< 0.001	61.5 ± 10.5	66.3 ± 9.2	< 0.001
Smoking history, n (%)	74 (27.8)	63 (51.6)	< 0.001	57 (24.8)	80 (50.6)	< 0.001
BMI, kg/m^2	24 ± 3.3	23.1 ± 3.4	0.020	24.3 ± 3.1	22.8 ± 3.5	< 0.001
**Spirometry**						
FVC, %pred	94.8 ± 16.2	78.4 ± 19.5	< 0.001	92.2 ± 15.6	85.9 ± 22.4	0.001
FEV1, %pred	106.4 ± 17.3	67.9 ± 21	< 0.001	99.7 ± 17.8	86.3 ± 32.6	< 0.001
FEV1/FVC, %	90.1 ± 6.8	68.1 ± 13	< 0.001	86.9 ± 9.5	77.8 ± 16.9	< 0.001
**PRM**						
PRM^{Emph} (%)	3.1 ± 3.7	11.5 ± 11.9	< 0.001	2.4 ± 4.4	10.7 ± 10	< 0.001
PRM^{fSAD} (%)	17 ± 15.9	28 ± 18.4	< 0.001	8.5 ± 5.5	37.9 ± 13.7	< 0.001
PRM^{Normal} (%)	77.3 ± 18.1	58.2 ± 25.3	< 0.001	86.1 ± 9.5	49.7 ± 17.9	< 0.001
**CT visual evaluation**						
Emphysema, n (%)	28 (10.5)	56 (45.9)	< 0.001	11 (4.8)	73 (46.2)	< 0.001
Tree-in-bud sign, n (%)	44 (16.5)	46 (37.7)	< 0.001	29 (12.6)	61 (38.6)	< 0.001
BWT, n (%)	10 (3.8)	44 (36.1)	< 0.001	17 (7.4)	37 (23.5)	< 0.001
Bronchial dilation, n (%)	82 (30.8)	28 (23)	0.110	64 (27.8)	46 (29.1)	0.782
**CT quantitative evaluation**						
AWT-Pi10	3.3 ± 0.8	3.9 ± 0.8	< 0.001	3.5 ± 0.8	3.5 ± 0.8	0.686
Branch count, ea	280 ± 81	233 ± 96	<	259 ± 81	274 ± 99	0.108

			0.001			
Lumen area, mm^2	9.6 ± 2.5	8.9 ± 2.5	0.011	9.2 ± 2.5	9.7 ± 2.6	0.108
Wall area, mm^2	16.2 ± 4.4	18.3 ± 4.8	< 0.001	16.8 ± 4.7	16.9 ± 4.5	0.837
Wall area, %	53.1 ± 9	60.1 ± 9.8	< 0.001	55.4 ± 9.4	55.1 ± 10.4	0.806
BV1/TBV	0.04 ± 0.01	0.03 ± 0.01	< 0.001	0.04 ± 0.01	0.03 ± 0.01	0.002
PV_6 CSA, mm^2	3.3 ± 1.2	3.6 ± 1.4	0.008	3.4 ± 1.3	3.3 ± 1.3	0.636
PV_9 CSA, mm^2	4 ± 1.3	4.3 ± 1.3	0.085	4.2 ± 1.3	4 ± 1.3	0.054
PV_{15} CSA, mm^2	6.2 ± 1.7	6.4 ± 1.8	0.359	6.5 ± 1.7	5.9 ± 1.7	< 0.001
PV_{21} CSA, mm^2	7.3 ± 1.9	7.5 ± 2.1	0.320	7.6 ± 2	7 ± 2	0.003

Note.—Data are mean ± SD unless indicated otherwise. HRCT = high-resolution computed tomography, PRM = parametric response mapping, BMI = body mass index, FVC = forced vital capacity, FEV1 = forced expiratory volume in the first second, PRM^{Emph} = the volume percentage of emphysema in PRM, PRM^{fSAD} = the volume percentage of functional small airway disease in PRM, PRM^{Normal} = the volume percentage of normal area in PRM, BWT = bronchial wall thickening, AWT = airway wall thickness, Pi10 = square root of wall area for airway with internal perimeter of 10 mm, BV1 = blood volume with cross-sectional area less than 1 mm^2, TBV = total blood volume, PV_n = pulmonary vessel at n mm from pleura, CSA = cross-sectional areas.

Supplementary Table E3: Spirometry Indices for Defining Small Airway Dysfunction (Four Subgroups)

	Spirometry (+) PRM (+) (n=75)	Spirometry (+) PRM (-) (n=47)	Spirometry (-) PRM (+) (n=83)	Spirometry (-) PRM (-) (n=183)	*P* Value
FEF 50%, %pred	29.9 ± 17.4	50.5 ± 16.2*	105.4 ± 28.1*#	104.7 ± 26.9*#^	< 0.001
FEF 75%, %pred	32.3 ± 18	43.2 ± 15.2*	137.1 ± 74.2*#	119.9 ± 51.4*#	< 0.001
MMEF, %pred	29.3 ± 16.8	47.1 ± 13.7*	108.2 ± 34.7*#	105.4 ± 27.1*#	< 0.001

Note.—Data described as mean ± SD. PRM = parametric response mapping, FEF 50% = forced expiratory flow at 50% of FVC, FEF 75% = forced expiratory flow at 75% of FVC, MMEF = maximum mid expiratory flow at 25-75% of FVC, FVC = forced vital capacity.

* $P < 0.05$, compared to Spirometry (+) PRM (+); # $P < 0.05$, compared to Spirometry (+) PRM (-); ^ *P* <

0.05, compared to Spirometry (-) PRM (+)

Supplementary Table E4: Spirometry Indices for Defining Small Airway Dysfunction

	Spirometry (-) (n=266)	Spirometry (+) (n=122)	*P* Value	PRM (-) (n=230)	PRM (+) (n=158)	*P* Value
FEF 50%, %pred	104.9 ± 27.3	37.8 ± 19.7	< 0.001	93.6 ± 33.3	69.5 ± 44.6	< 0.001
FEF 75%, %pred	125.2 ± 59.8	36.5 ± 17.7	< 0.001	104.2 ± 55.7	87.1 ± 76	0.011
MMEF, %pred	106.2 ± 29.7	36.1 ± 17.9	< 0.001	93.4 ± 34.3	70.7 ± 48.2	< 0.001

Note.—Data described as mean ± SD. PRM = parametric response mapping, FEF 50% = forced expiratory flow at 50% of FVC, FEF 75% = forced expiratory flow at 75% of FVC, MMEF = maximum mid expiratory flow at 25-75% of FVC, FVC = forced vital capacity.

Supplementary Table E5: Airflow Limitation Characteristics of Participants

	Spirometry (-) (n=266)	Spirometry (+) (n=122)	PRM (-) (n=230)	PRM (+) (n=158)
PRISm	7 (2.6)	25 (20.5)	20 (8.7)	12 (7.6)
GOLD 1-2	0 (0)	44 (36.1)	11 (4.8)	33 (20.9)
GOLD 3-4	0 (0)	25 (20.5)	1 (0.4)	24 (15.2)

Note.—Data are presented as numbers, with percentages in parentheses. PRM = parametric response mapping, PRISm = preserved ratio and impaired spirometry, GOLD = global initiative for chronic obstructive lung disease.

Supplementary Table E6: Visual Assessment of HRCT in Participants (Four Subgroups)

	Spirometry (+) PRM (+) (n=75)	Spirometry (+) PRM (-) (n=47)	Spirometry (-) PRM (+) (n=83)	Spirometry (-) PRM (-) (n=183)	*P* Value
Mucus plug, n (%)	17 (22.7)	4 (8.5)	8 (9.6)	5 (2.7)*	< 0.001
PA diameter ≥2.9 cm, n (%)	8 (10.7)	10 (21.3)	10 (12)	37 (20.2)	0.134
ILA, n (%)	5 (6.7)	5 (10.6)	10 (12)	12 (6.6)	0.411

Note.—Data are presented as numbers, with percentages in parentheses. PRM = parametric response mapping, PA = pulmonary artery, ILA = interstitial lung abnormalities.

* *P* < 0.05, compared to Spirometry (+) PRM (+); # *P* < 0.05, compared to Spirometry (+) PRM (-); ^ *P* < 0.05, compared to Spirometry (-) PRM (+)

Supplementary Table E7: Visual Assessment of HRCT in Participants

	Spirometry (-) (n=266)	Spirometry (+) (n=122)	*P* Value	PRM (-) (n=230)	PRM (+) (n=158)	*P* Value
Mucus plug, n (%)	13 (4.9)	21 (17.2)	< 0.001	9 (3.9)	25 (15.8)	< 0.001
PA diameter ≥2.9 cm, n (%)	47 (17.7)	18 (14.8)	0.475	47 (20.4)	18 (11.4)	0.019
ILA, n (%)	22 (8.3)	10 (8.2)	0.980	17 (7.4)	15 (9.5)	0.460

Note.—Data are presented as numbers, with percentages in parentheses. PRM = parametric response mapping, PA = pulmonary artery, ILA = interstitial lung abnormalities.

Supplementary Table E8: Quantitative HRCT Parameters of Airway at Whole-Lung Level in Participants (Four Subgroups)

	Spirometry (+) PRM (+) (n=75)	Spirometry (+) PRM (-) (n=47)	Spirometry (-) PRM (+) (n=83)	Spirometry (-) PRM (-) (n=183)	*P* Value
Lumen diameter, mm	3 ± 0.4	2.9 ± 0.4	3.2 ± 0.4*#	3.1 ± 0.4*#^	< 0.001
Wall thickness, mm	1.1 ± 0.3	1.1 ± 0.3	0.9 ± 0.2*#	1 ± 0.2*^	< 0.001
Branch length, mm	12.1 ± 1.4	11.7 ± 1.3	11.2 ± 1.1*	11.2 ± 1*	< 0.001
Airway area, mm^2	27.9 ± 5.9	26.2 ± 5.3	25.4 ± 4.3*	26 ± 5.3*	0.018

Note.—Data described as mean ± SD. HRCT = high-resolution computed tomography, PRM = parametric response mapping.

* $P < 0.05$, compared to Spirometry (+) PRM (+); # $P < 0.05$, compared to Spirometry (+) PRM (-); ^ $P < 0.05$, compared to Spirometry (-) PRM (+)

Supplementary Table E9: Quantitative HRCT Parameters of Airway at Whole-Lung Level in Participants

	Spirometry (-) (n=266)	Spirometry (+) (n=122)	*P* Value	PRM (-) (n=230)	PRM (+) (n=158)	*P* Value
Lumen diameter, mm	3.2 ± 0.4	3 ± 0.4	< 0.001	3.1 ± 0.4	3.1 ± 0.4	0.494
Wall thickness, mm	0.9 ± 0.2	1.1 ± 0.3	< 0.001	1 ± 0.3	1 ± 0.3	0.863
Branch length, mm	11.2 ± 1.1	11.9 ± 1.4	< 0.001	11.3 ± 1.1	11.6 ± 1.3	0.015
Airway area, mm^2	25.8 ± 5	27.2 ± 5.7	0.014	26.1 ± 5.3	26.6 ± 5.2	0.342

Note.—Data described as mean ± SD. HRCT = high-resolution computed tomography, PRM = parametric response mapping.

Supplementary Table E10: Quantitative HRCT Parameters of 5th to 8th Generation Airways in Participants (Four Subgroups)

	Spirometry (+) PRM (+) (n=75)	Spirometry (+) PRM (-) (n=47)	Spirometry (-) PRM (+) (n=83)	Spirometry (-) PRM (-) (n=183)	*P* Value
**5th generation**					
AWT-Pi10	5.4 ± 1.3	5.3 ± 1.6	4.6 ± 1.6*#	4.8 ± 1.4*#	0.002
Branch count, ea	11.5 ± 4.5	11.7 ± 3.9	12.8 ± 4.1	13.7 ± 3.8*#	< 0.001
Lumen area, mm^2	25.4 ± 16.1	21.4 ± 9.3	26.7 ± 10.9#	24.2 ± 11.4	0.105
Wall area, mm^2	56.3 ± 21.7	51.4 ± 14.6	52.3 ± 16.4	54.5 ± 20.4	0.442
Wall area, %	69.5 ± 9.1	68.8 ± 9.1	63.2 ± 7.8*#	66.1 ± 7.6*#^	< 0.001
Airway diameter, mm	9.6 ± 1.9	9 ± 1.2	9.4 ± 1.3	9.3 ± 1.5	0.218
**6th generation**					
AWT-Pi10	4.7 ± 1.1	4.3 ± 1*	3.6 ± 1*#	4.1 ± 0.9*^	< 0.001
Branch count, ea	22.8 ± 7.5	23.6 ± 6.6	26.3 ± 6.8*#	27.8 ± 6.1*#	< 0.001
Lumen area, mm^2	16.9 ± 11.9	13.8 ± 8.5	17.8 ± 8.2	14.9 ± 7.6^	0.018
Wall area, mm^2	38.6 ± 20.5	29.2 ± 11*	33.1 ± 13.8	32.7 ± 15.1	0.008
Wall area, %	67 ± 9.1	64.8 ± 9.9	57.2 ± 8.1*#	61.5 ± 8.4*#^	< 0.001
Airway diameter, mm	7.5 ± 1.9	6.6 ± 1.2*	7.2 ± 1.3	6.9 ± 1.2	0.002
**7th generation**					
AWT-Pi10	4.2 ± 0.9	4 ± 1	3.3 ± 0.8*#	3.6 ± 0.8*#^	< 0.001
Branch count, ea	35.6 ± 12.5	36.6 ± 11.2	43 ± 9.7*#	44.5 ± 10.2*#	< 0.001
Lumen area, mm^2	9.9 ± 5.2	8.2 ± 4.1	12.2 ± 6.6*#	9.6 ± 4.3^	< 0.001
Wall area, mm^2	22.7 ± 10.8	17.5 ± 5.7*	20.1 ± 12.8	17.6 ± 6.5*	< 0.001
Wall area, %	63.7 ± 9.5	61.7 ± 10.1	52.6 ± 8.3*#	56.4 ± 8.4*#^	< 0.001
Airway diameter, mm	5.7 ± 1.2	5.1 ± 0.8*	5.6 ± 1.1	5.2 ± 0.8*	<

					0.001
**8th generation**					
AWT-Pi10	3.8 ± 0.8	3.8 ± 1.1	3.1 ± 0.7*#	3.3 ± 0.7*#^	< 0.001
Branch count, ea	42 ± 16.5	41.6 ± 16.6	52.5 ± 13.6*#	51.4 ± 14.9*#	< 0.001
Lumen area, mm^2	7.6 ± 4.1	6.7 ± 3.1	8.9 ± 3.3#	7.6 ± 2.8^	0.001
Wall area, mm^2	15.9 ± 8.6	13.5 ± 6.6	12.6 ± 5.5*	12 ± 5.1*	< 0.001
Wall area, %	60.5 ± 9.3	58.7 ± 10.1	49.9 ± 8.6*#	53.2 ± 8.7*#^	< 0.001
Airway diameter, mm	4.8 ± 1	4.5 ± 0.7	4.7 ± 0.7	4.5 ± 0.6	0.025

Note.—Data described as mean ± SD. HRCT = high-resolution computed tomography, PRM = parametric response mapping, AWT = airway wall thickness, Pi10 = square root of wall area for airway with internal perimeter of 10 mm.

* *P* < 0.05, compared to Spirometry (+) PRM (+); # *P* < 0.05, compared to Spirometry (+) PRM (-); ^ *P* < 0.05, compared to Spirometry (-) PRM (+)

Supplementary Table E11: Quantitative HRCT Parameters of 5th to 8th Generation Airways in Participants

	Spirometry (-) (n=266)	Spirometry (+) (n=122)	*P* Value	PRM (-) (n=230)	PRM (+) (n=158)	*P* Value
**5th generation**						
AWT-Pi10	4.7 ± 1.5	5.4 ± 1.4	< 0.001	4.9 ± 1.5	5 ± 1.5	0.764
Branch count, ea	13.4 ± 3.9	11.6 ± 4.3	< 0.001	13.3 ± 3.9	12.2 ± 4.3	0.011
Lumen area, mm^2	25 ± 11.3	23.8 ± 14	0.421	23.7 ± 11.1	26.1 ± 13.6	0.056
Wall area, mm^2	53.8 ± 19.3	54.4 ± 19.3	0.801	53.9 ± 19.4	54.2 ± 19.1	0.896
Wall area, %	65.2 ± 7.7	69.2 ± 9.1	< 0.001	66.7 ± 7.9	66.2 ± 9	0.558
Airway diameter, mm	9.3 ± 1.4	9.4 ± 1.7	0.632	9.2 ± 1.4	9.5 ± 1.6	0.117
**6th generation**						
AWT-Pi10	3.9 ± 1	4.5 ± 1.1	< 0.001	4.1 ± 1	4.1 ± 1.2	0.889

Branch count, ea	27.3 ± 6.3	23.1 ± 7.2	< 0.001	26.9 ± 6.4	24.7 ± 7.4	0.002
Lumen area, mm^2	15.8 ± 7.9	15.7 ± 10.8	0.919	14.6 ± 7.8	17.4 ± 10.1	0.004
Wall area, mm^2	32.8 ± 14.7	34.9 ± 18	0.222	31.9 ± 14.4	35.7 ± 17.4	0.026
Wall area, %	60.2 ± 8.6	66.1 ± 9.5	< 0.001	62.2 ± 8.8	61.8 ± 9.9	0.690
Airway diameter, mm	7 ± 1.3	7.1 ± 1.7	0.335	6.8 ± 1.2	7.3 ± 1.6	0.002
**7th generation**						
AWT-Pi10	3.5 ± 0.8	4.1 ± 0.9	< 0.001	3.7 ± 0.8	3.7 ± 1	0.467
Branch count, ea	44 ± 10	36 ± 12	< 0.001	42.8 ± 10.9	39.5 ± 11.7	0.004
Lumen area, mm^2	10.4 ± 5.2	9.2 ± 4.8	0.035	9.3 ± 4.3	11.1 ± 6	0.002
Wall area, mm^2	18.4 ± 9	20.7 ± 9.5	0.023	17.6 ± 6.4	21.3 ± 12	< 0.001
Wall area, %	55.2 ± 8.6	62.9 ± 9.8	< 0.001	57.5 ± 9	57.8 ± 10.5	0.757
Airway diameter, mm	5.3 ± 0.9	5.5 ± 1.1	0.216	5.2 ± 0.8	5.6 ± 1.2	< 0.001
**8th generation**						
AWT-Pi10	3.2 ± 0.7	3.8 ± 0.9	< 0.001	3.4 ± 0.8	3.4 ± 0.8	0.961
Branch count, ea	51.8 ± 14.5	41.8 ± 16.5	< 0.001	49.4 ± 15.7	47.5 ± 15.9	0.251
Lumen area, mm^2	8 ± 3	7.2 ± 3.8	0.024	7.5 ± 2.9	8.3 ± 3.8	0.021
Wall area, mm^2	12.2 ± 5.2	14.9 ± 7.9	0.001	12.3 ± 5.4	14.2 ± 7.3	0.006
Wall area, %	52.2 ± 8.8	59.8 ± 9.6	< 0.001	54.4 ± 9.2	54.9 ± 10.4	0.602
Airway diameter, mm	4.6 ± 0.7	4.7 ± 0.9	0.179	4.5 ± 0.7	4.8 ± 0.9	0.006

Note.—Data described as mean ± SD. HRCT = high-resolution computed tomography, PRM = parametric response mapping, AWT = airway wall thickness, Pi10 = square root of wall area for airway with internal perimeter of 10 mm.

Supplementary Table E12. Quantitative HRCT Parameters of Pulmonary Vascular Blood Flow Volume in Participants (Four Subgroups)

	Spirometry (+) PRM (+) (n=75)	Spirometry (+) PRM (-) (n=47)	Spirometry (-) PRM (+) (n=83)	Spirometry (-) PRM (-) (n=183)	*P* Value
BV1, mm^3	6.2 ± 3.5	6.8 ± 3	7.6 ± 3.5*	7.3 ± 3*	0.02
BV1/TBV	0.03 ± 0.01	0.04 ± 0.01*	0.04 ± 0.01*	0.04 ± 0.01*	< 0.001
BV5, mm^3	87.9 ± 29.2	84.1 ± 21.9	89 ± 23.9	83.7 ± 22.7	0.314
BV5/TBV	0.42 ± 0.06	0.44 ± 0.04*	0.44 ± 0.05*	0.44 ± 0.05*	0.02
BV10, mm^3	158.2 ± 48.3	145.8 ± 34.4	154.1 ± 36.3	145.5 ± 35.5	0.061
BV10/TBV	0.76 ± 0.04	0.77 ± 0.04	0.76 ± 0.04	0.76 ± 0.04	0.33
TBV, mm^3	209.4 ± 63.9	190.4 ± 46.4*	203.1 ± 49.2	191.2 ± 47.1*	0.034

Note.—Data described as mean ± SD. HRCT = high-resolution computed tomography, PRM = parametric response mapping, BV1 = blood volume with cross-sectional area less than 1 mm^2, BV5 = blood volume with cross-sectional area less than 5 mm^2, BV10 = blood volume with cross-sectional area less than 10 mm^2, TBV = total blood volume.

* $P < 0.05$, compared to Spirometry (+) PRM (+); # $P < 0.05$, compared to Spirometry (+) PRM (-); ^ $P < 0.05$, compared to Spirometry (-) PRM (+)

Supplementary Table E13. Quantitative HRCT Parameters of Pulmonary Vascular Blood Flow Volume in Participants

	Spirometry (-) (n=266)	Spirometry (+) (n=122)	*P* Value	PRM (-) (n=230)	PRM (+) (n=158)	*P* Value
BV1, mm^3	7.4 ± 3.1	6.4 ± 3.3	0.004	7.2 ± 3	6.9 ± 3.5	0.483
BV1/TBV	0.04 ± 0.01	0.03 ± 0.01	< 0.001	0.04 ± 0.01	0.03 ± 0.01	0.002
BV5, mm^3	85.3 ± 23.2	86.4 ± 26.6	0.682	83.8 ± 22.5	88.5 ± 26.5	0.062
BV5/TBV	0.44 ± 0.05	0.43 ± 0.05	0.076	0.44 ± 0.05	0.43 ± 0..05	0.066
BV10, mm^3	148.2 ± 35.9	153.4 ± 43.7	0.252	145.6 ± 35.2	156 ± 42.3	0.008
BV10/TBV	0.76 ± 0.04	0.76 ± 0.04	0.635	0.76 ± 0.04	0.76 ± 0.04	0.191
TBV, mm^3	194.9 ± 48	202.1 ± 58.3	0.237	191 ± 46.9	206.1 ± 56.6	0.005

Note.—Data described as mean ± SD. HRCT = high-resolution computed tomography, PRM = parametric response mapping, BV1 = blood volume with cross-sectional area less than 1 mm^2, BV5 = blood volume with cross-sectional area less than 5 mm^2, BV10 = blood volume with cross-sectional area less than 10 mm^2, TBV = total blood volume.

Supplementary Table E14. Other Quantitative HRCT Parameters of Pulmonary Vessels in Participants (Four Subgroups)

	Spirometry (+) PRM (+) (n=75)	Spirometry (+) PRM (-) (n=47)	Spirometry (-) PRM (+) (n=83)	Spirometry (-) PRM (-) (n=183)	*P*
PV_6 total surface area, mm^2	253209 ± 46467	214912 ± 45406*	241879 ± 40440#	211413 ± 45062*^	< 0.001
PV_6 total CSA, mm^2	3381 ± 1516	4045 ± 1485*	3606 ± 1446	4210 ± 1449*^	< 0.001
PV_6 diameter, mm	1.9 ± 0.3	1.8 ± 0.2	1.8 ± 0.2*	1.8 ± 0.2*	< 0.001
Number of PV_6 with CSA under $5mm^2$, ea	825 ± 424	1025 ± 410*	1093 ± 419*	1122 ± 397*	< 0.001
Number of PV_6 with CSA under $5mm^2$, %	80.4 ± 8.4	83.3 ± 5.7	85.2 ± 6.1*	84.4 ± 5.7*	< 0.001
PV_6 total number, ea	1003 ± 473	1218 ± 446*	1272 ± 462*	1316 ± 434*	< 0.001
PV_9 total surface area, mm^2	210009 ± 40458	177974 ± 41640*	201405 ± 36092#	172792 ± 38412*^	< 0.001
PV_9 total CSA, mm^2	4417 ± 1675	4830 ± 1470	4530 ± 1531	4862 ± 1419*	0.104
PV_9 diameter, mm	2.1 ± 0.2	2.1 ± 0.2	2 ± 0.2*#	2.1 ± 0.2^	0.003
Number of PV_9 with CSA under $5mm^2$, ea	819.2 ± 310.4	874.6 ± 270	1004 ± 298.9*#	919 ± 283.8*^	0.001
Number of PV_9 with CSA under $5mm^2$, %	75.2 ± 8.1	75.6 ± 7.6	78.9 ± 6.2*#	75.8 ± 7.4^	0.004
PV_9 total number, ea	1078 ± 360	1149 ± 306	1266 ± 342*	1199 ± 315*	0.004
PV_{15} total surface area, mm^2	147260 ± 38547	120423 ± 37016*	139191 ± 32753#	118540 ± 38260*^	< 0.001
PV_{15} total CSA, mm^2	4441 ± 1703	4305 ± 1556	4079 ± 1315	4128 ± 1372	0.347
PV_{15} diameter, mm	2.6 ± 0.2	2.7 ± 0.2	2.6 ± 0.2*#	2.7 ± 0.2^	0.001
Number of PV_{15} with CSA under $5mm^2$, ea	407 ± 129	353 ± 108*	428 ± 131#	352 ± 126*^	< 0.001
Number of PV_{15} with CSA under $5mm^2$, %	55.9 ± 8.3	54.1 ± 7.8	57.8 ± 8.6#	53.2 ± 8.6*^	< 0.001
PV_{15} total number, ea	723 ± 182	648 ± 165*	735 ± 172#	649 ± 175*^	< 0.001
PV_{21} total surface area, mm^2	92456 ± 31585	67733 ± 28068*	81363 ± 23484	64525 ± 25108*	< 0.001

PV_{21} total CSA, mm^2	2999 ± 1280	2606 ± 1122*	2634 ± 892*	2490 ± 989*	0.006
PV_{21} diameter, mm	3 ± 0.2	3 ± 0.2	2.9 ± 0.3*#	3 ± 0.2^	0.011
Number of PV_{21} with CSA under $5mm^2$, ea	180.2 ± 65.6	144.9 ± 60.6*	178.6 ± 64.1#	142.3 ± 59.9*^	< 0.001
Number of PV_{21} with CSA under $5mm^2$, %	43.2 ± 7.2	42.3 ± 7.2	44.4 ± 8.5	41.7 ± 6.9^	0.041
PV_{21} total number, ea	412.1 ± 120.2	337 ± 117.6*	396.9 ± 107#	334.6 ± 113*^	< 0.001

Note.—Data described as mean ± SD. HRCT = high-resolution computed tomography, PRM = parametric response mapping, PV_n = pulmonary vessel at n mm from pleura, CSA = cross-sectional areas.
* $P < 0.05$, compared to Spirometry (+) PRM (+); # $P < 0.05$, compared to Spirometry (+) PRM (-); ^ $P < 0.05$, compared to Spirometry (-) PRM (+)

Supplementary Table E15. Other Quantitative HRCT Parameters of Pulmonary Vessels in Participants

	Spirometry (-) (n=266)	Spirometry (+) (n=122)	*P*	PRM (-) (n=230)	PRM (+) (n=158)	*P*
PV_6 total surface area, mm^2	220920 ± 45833	238455 ± 49543	0.001	212128 ± 45055	247257 ± 43636	< 0.001
PV_6 total CSA, mm^2	4022 ± 1473	3637 ± 1533	0.019	4177 ± 1455	3500 ± 1480	< 0.001
PV_6 diameter, mm	1.8 ± 0.2	1.9 ± 0.2	< 0.001	1.8 ± 0.2	1.8 ± 0.2	0.088
Number of PV_6 with CSA under $5mm^2$, ea	1113 ± 404	902 ± 428	< 0.001	1102 ± 401	966 ± 441	0.002
Number of PV_6 with CSA under $5mm^2$, %	84.6 ± 5.9	81.5 ± 7.6	< 0.001	84.2 ± 5.7	82.9 ± 7.6	0.080
PV_6 total number, ea	1302 ± 443	1086 ± 473	< 0.001	1296 ± 437	1145 ± 485	0.002
PV_9 total surface area, mm^2	181720 ± 39911	197668 ± 43649	< 0.001	173851 ± 39056	205490 ± 38346	< 0.001
PV_9 total CSA, mm^2	4759 ± 1460	4577 ± 1606	0.270	4856 ± 1426	4477 ± 1597	0.015
PV_9 diameter, mm	2.1 ± 0.2	2.1 ± 0.2	0.037	2.1 ± 0.2	2.1 ± 0.2	0.088
Number of PV_9 with CSA under $5mm^2$, ea	946 ± 291	841 ± 296	0.001	910 ± 281	916 ± 317	0.835
Number of PV_9 with CSA under	76.8 ± 7.2	75.4 ± 7.9	0.083	75.8 ± 7.4	77.2 ± 7.4	0.069

$5mm^2$, %						
PV_{9} total number, ea	1220 ± 325	1105 ± 341	0.002	1189 ± 314	1177 ± 362	0.723
PV_{15} total surface area, mm^2	124983 ± 37804	136921 ± 40019	0.005	118925 ± 37937	143021 ± 35735	< 0.001
PV_{15} total CSA, mm^2	4113 ± 1352	4388 ± 1643	0.108	4165 ± 1409	4251 ± 1518	0.566
PV_{15} diameter, mm	2.6 ± 0.2	2.7 ± 0.2	0.856	2.7 ± 0.2	2.6 ± 0.2	0.001
Number of PV_{15} with CSA under $5mm^2$, ea	375 ± 132	386 ± 124	0.441	352 ± 123	418 ± 130	< 0.001
Number of PV_{15} with CSA under $5mm^2$, %	54.6 ± 8.9	55.2 ± 8.1	0.525	53.3 ± 8.5	56.9 ± 8.5	< 0.001
PV_{15} total number, ea	676 ± 178	694 ± 179	0.346	649 ± 172	729 ± 177	< 0.001
PV_{21} total surface area, mm^2	69779 ± 25782	82931 ± 32490	< 0.001	65181 ± 25709	86629 ± 28092	< 0.001
PV_{21} total CSA, mm^2	2535 ± 961	2848 ± 1232	0.014	2514 ± 1016	2808 ± 1105	0.007
PV_{21} diameter, mm	2.9 ± 0.2	3 ± 0.2	0.522	3 ± 0.2	2.9 ± 0.2	0.012
Number of PV_{21} with CSA under $5mm^2$, ea	153.6 ± 63.4	166.6 ± 65.8	0.066	142.8 ± 59.9	179.4 ± 64.6	< 0.001
Number of PV_{21} with CSA under $5mm^2$, %	42.6 ± 7.5	42.8 ± 7.2	0.725	41.8 ± 6.9	43.8 ± 7.9	0.009
PV_{21} total number, ea	354 ± 114.7	383.2 ± 124.3	0.024	335.1 ± 113.7	404.1 ± 113.4	< 0.001

Note.—Data described as mean ± SD. HRCT = high-resolution computed tomography, PRM = parametric response mapping, PV_n = pulmonary vessel at n mm from pleura, CSA = cross-sectional areas.

Supplementary Table E16. Area Under the ROC Curve in the Predictive Model of SAD Defined by Spirometry

Variable	AUC	95% CI

**Clinical data**	0.691	0.633 to 0.749
Age	0.655	0.597 to 0.713
Male	0.612	0.563 to 0.662
**CT evaluation**	0.834	0.786 to 0.881
PRM^{Emph}	0.746	0.689 to 0.802
Tree-in-bud sign	0.607	0.559 to 0.656
BWT	0.659	0.615 to 0.703
Branch count	0.663	0.601 to 0.725
**Fitted model nomogram**	0.855	0.809 to 0.902

Note.—ROC = receiver operating characteristic, SAD = small airway dysfunction, AUC = area under the ROC curve, CI = confidence interval, PRM^{Emph} = the volume percentage of emphysema in PRM, PRM = parametric response mapping, BWT = bronchial wall thickening.

Supplementary Table E17. Area Under the ROC Curve in the Predictive Model of SAD Defined by PRM

Variable	AUC	95% CI
**Clinical data**	0.738	0.688 to 0.789
Age	0.628	0.573 to 0.683
Male	0.648	0.602 to 0.694
BMI	0.638	0.581 to 0.695
**CT evaluation**	0.766	0.715 to 0.817
Tree-in-bud sign	0.63	0.586 to 0.674
Emphysema	0.707	0.666 to 0.749
BV1/TBV	0.593	0.535 to 0.652
**Fitted model nomogram**	0.808	0.763 to 0.852

Note.—ROC = receiver operating characteristic, SAD = small airway dysfunction, PRM = parametric response mapping, AUC = area under the ROC curve, CI = confidence interval, BMI = body mass index, BV1 = blood volume with cross-sectional area less than 1 mm^2; TBV = total blood volume.
